# Therapeutic Modalities for Asthma, COPD, and Pathogenesis of COVID-19: Insights from the Special Issue

**DOI:** 10.3390/jcm11154525

**Published:** 2022-08-03

**Authors:** Sukhwinder Singh Sohal

**Affiliations:** Respiratory Translational Research Group, Department of Laboratory Medicine, School of Health Sciences, College of Health and Medicine, University of Tasmania, Launceston, TAS 7248, Australia; sukhwinder.sohal@utas.edu.au

The human lung is a vital organ, which is vulnerable to outside insults and injuries. Nothing else matters when you cannot breathe. The pharmacological management of chronic lung disease is a rapidly growing field. Some advances have been made in unravelling the mechanisms underlying the pathogenesis of asthma, COPD, and other chronic lung disorders, such as interstitial lung diseases (ILD). However, there is still a lack of clinical translation of various in vivo and in vitro studies. There is a serious need to understand the nature and mechanism of action of the current pharmacological therapy for chronic lung disease. Many could be repurposed, but it is vital to understand mechanisms both at a disease and therapy level to establish a targeted approach. What are the challenges in the treatment of patients with COPD and asthma? Which disease mechanisms respond to current therapeutics, and which do not? How can we optimize drug therapy in COPD and asthmatic patients? Managing these patients has become even more challenging as COVID-19 continues its grasp in 2022, although vaccine development has been helpful in improving immunity and protecting the community. Considering these important research questions, the *Journal of Clinical Medicine (JCM)* recently launched a Special Issue targeting these areas, titled Pharmacology and Therapeutic of Asthma and COPD. The issue has been a great success since being released and has published five manuscripts so far with others in the pipeline. These include four original research articles and one review article. In this Editorial, I would like to share insights from these articles with the readership in the setting of the current literature in the field.

Jung et al. reported, using a large Korean cohort of 4066 COVID-19 patients, that neither mild nor severe asthma was associated with the severity or mortality of COVID-19, even after adjusting for variables [1]. A subgroup analysis with smoking history or current smoking showed that mild asthma was associated with COVID-19, which may reflect asthma–COPD overlap (ACO). For patients with COPD, however, a severe form of disease was a significant risk factor for developing severe COVID-19 and mortality. In underweight patients, mild COPD was associated with COVID-19. The study has limitations though, as asthma and COPD were not defined by the international standards; hence, it is hard to tease out the relationship between asthma, COPD and COVID-19 based on the information provided by the authors. Smoking has been suggested as one of the major risk factors for the development of COVID-19 in smokers and COPD [2,3]. The current study could not provide detailed analysis highlighting such associations, which might be partly due to the availability of accurate smoking status or patients being on inhaled corticosteroids or other medications. There are certainly smokers and ex-smokers in the cohort but there is no clear information provided in the analysis. However, the UK Biobank Cohort study indicated a causal effect of smoking in the risk of severe COVID-19 [4]. There is now substantial evidence available from clinical pathological studies using human samples from smokers and patients with COPD, providing mechanistic links between smoking and COVID-19. In some parts of the world, especially India, mucormycosis, also known as the black fungus, has been reported [5]. Authors suggested that the excessive use of corticosteroids in the treatment of COVID-19 and immunosuppression by the virus has led to such opportunistic infections [6].

In March 2020, our research group was first to provide the evidence that the SARS-CoV-2 receptor ACE2 is elevated in the airway epithelium, type-2 pneumocytes and alveolar macrophages of smokers and patients with COPD [7], which, since then, has been confirmed by several studies in the literature [8,9,10]. We further reported, in another study, that in addition to ACE2, cofactors, such as TMPRSS2 and Furin, are also highly expressed on the small airway epithelium, alveolar macrophages and type-2 pneumocytes [11]. We found similar expression in the primary small airway epithelial cells too. Interestingly, there was a significant increase in type-2 pneumocytes in smokers and patients with COPD compared to normal never-smoking controls. Increased type-2 pneumocytes suggest that these patients are vulnerable to developing post-COVID-19 interstitial pulmonary fibrosis or fibrosis in general. We found similar changes in patients with ILD, such as lymphangioleiomyomatosis and idiopathic pulmonary fibrosis (IPF) [12,13]. There could be a silently developing interstitial pathology in smokers and patients with COPD. This was the first study to report an increase in type-2 pneumocytes in these patients [11]. Further, we also reported that endocytic vacuoles, such as early endosome antigen-1, late endosome marker RAB7, cathepsin-L and lysosomal-associated membrane protein-1, as lysosome markers, also increase in the airways of smokers and patients with COPD [14]. This study indicated that smoking not only upregulates ACE2 for viral binding but also creates a highly conducive environment for the virus to thrive. The inflammatory environment created by M1/M2 macrophages in smokers and COPD further exaggerate this lethal microbial pathogenesis [15,16]. In line with this, a recent study by Hönzke and colleagues reported that severe lung injury in COVID-19 likely results from a macrophage-mediated immune response rather than direct viral injury of the alveolar areas [17]. This suggests that smokers and patients with COPD are already primed for COVID-19-related lung damage. In patients with asthma, Wark and colleagues reported lower levels of ACE2 but it was positively associated with older age and male gender [18]. COPD still had higher ACE2 levels compared to asthmatics [19]. This may be the reason for the lack of representation of asthmatics with COVID-19 complications, as observed by Jung et al. in a Korean cohort [1].

Like smoking, vaping has also been suggested as a major risk factor for increasing susceptibility to SARS-CoV-2 and development of COVID-19 [20,21,22,23], as reported by McAlinden et al. in this Special Issue [24]. In this study, we observed that condensates generated from electronic cigarettes increased the ACE2 expression on both BEAS2-B and primary small airway epithelial cells in a similar manner to smoking. We also observed that vaping increased cytotoxicity and compromised membrane integrity, as measured by CCK-8 and LDH assays. The study also suggested that e-liquids with and without flavours or nicotine are toxic to cells; with flavour, toxicity increases though. Cell death was observed for nicotine-treated cells. This is the first study to utilize electronic-cigarette aerosol condensates, novel and developed in our laboratory, for investigating the effects of vaping on human airway epithelial cells. We previously reported electronic cigarettes and the heat-not-burn device IQOS increases inflammation (CXCL8), extracellular matrix release (collagen 1 and fibronectin) and mitochondrial dysfunction [25,26]. A recent study also showed that vaping leads to dysfunctional immunity, as we see in smokers and patients with COPD [27,28].

The next study, by Cerdán-de-las-Heras and colleagues, takes a rehabilitation therapy angle for the management of COPD [29]. In a randomized, non-inferiority study, authors evaluated the effect of a tele-rehabilitation program compared to standard rehabilitation on COPD. They screened 95 COPD patients in total. The tele-rehabilitation included physiotherapist video/chat consultations and workout sessions, with a virtual autonomous physiotherapist agent. Data were collected at baseline, 8 weeks, 3 and 6 months. The authors observed that there is better adherence, safety, and patient satisfaction in the tele-rehabilitation intervention arm. In COVID-19 times, this has become a promising alternative for patients with COPD and a better alternative in the long run. However, more studies with larger cohorts and severity of disease are needed.

Continuing the therapeutic theme, Huang and colleagues investigated the long-term efficacy of an anti-IgE antibody, Omalizumab, in a cohort of patients with severe allergic asthma [30]. Omalizumab comes under the class of medications called monoclonal antibodies [31,32,33]. A number of studies has investigated the long-term benefits of Omalizumab but hardly any studies comparing booster dose versus maintenance dose over time and clinical benefits [34,35,36]. The authors monitored this for 12 months, with a boost of Omalizumab treatment for the first 4 months, in a cohort of 124 patients with severe allergic asthma. This was the first study to compare the long-term efficacy between continuous use and 4-month boosts of Omalizumab in adult patients with severe allergic asthma. Their study demonstrated that continuous use of Omalizumab is beneficial compared to a short-term booster dose and long-term outcomes are worse on a short-term treatment. This study also found that small airway dysfunction decreased, and exacerbations increased at the 12-month follow-up when Omalizumab was boosted for 4 months. However, studies with larger cohorts, small airway dysfunction measuring techniques, such as forced oscillation technique [37], and a comparison to a paediatric population are needed. Mechanistic studies are also needed to validate these interactions.

The final article in this Issue raises the role of adjunct therapy in COPD [38]. The authors discussed the benefits and limitations for adding other therapies to routine COPD management. These included therapeutic modalities, such as roflumilast, macrolides, mucolytics, vitamin D supplementation, oral corticosteroids, n-acetylcysteine and nebulized hypertonic saline. Roflumilast is a phosphodiesterase-4 inhibitor, which has some anti-inflammatory properties. However, inflammation in COPD is a true paradox and there is huge variability with severity of disease [39,40]. We showed that mild to moderate COPD is associated with a lack of key immune cells in the airway wall, with macrophages and CD8 cells as the prominent cell types [15,41]. Milara J et al. showed that roflumilast can inhibit the process of epithelial to mesenchymal transition (EMT) in smokers with COPD [42]; the same group further reported that the addition of simvastatin enhanced the ability of roflumilast to inhibit EMT activity [43]. Hence, statins could be another adjunct therapy that could be added to COPD management, which may protect these patients from lung cancer development [44] or small airway fibrosis [45] by inhibiting EMT [46]. In 2010, I was first to report that EMT is an active process in smokers and patients with COPD [47,48] and inhaled fluticasone propionate has the potential to inhibit such changes [49]. However, reticular basement membrane hypervascularity did not change post-treatment [50]. Roflumilast and statins along with inhaled corticosteroids may have beneficial effects for COPD patients, especially for EMT-related changes [51]. Hybrid EMT has also been suggested in COPD and patients with IPF [52]. Endothelial-to-mesenchymal transition (EndMT) is a similar process to EMT, and these drugs may have similar effects on EndMT as well, but further work is warranted [53,54,55,56,57,58,59,60]. Oral corticosteroids are yet to be tested for their efficacy for such effects. Azithromycin, in addition to its efficacy as a macrolide and anti-inflammatory agent [61,62,63,64], has also been shown to prevent EMT changes [65,66,67,68,69].

Chronic bacterial colonization is a major issue in smokers and patients with COPD [70]. They can also be affected with viral infections, such as *Rhinovirus,* and more recently, SARS-CoV-2 (COVID-19) [71,72,73]. From an infection point of view, it is important to understand how these microbes get access to the lungs [74,75,76]. Highlighting this association, Grigg et al. showed that platelet-activating factor receptor (PAFR) is upregulated in response to cigarette smoking and is responsible for the adhesion of *Streptococcus pneumoniae* to lower airway cells [77]. The same group also reported similar changes in response to electronic-cigarette exposure or vaping [78]. We have further shown that PAFR increases in the small and large airways of both smokers and patients with COPD [16,70,71,79]. Similar observations were made for the *Rhinovirus* adhesion receptor, intercellular adhesion molecule-1 (ICAM-1) [16,70,71,79]. Inhaled corticosteroids could increase the risk for pneumonia, so a careful management is needed when prescribing these [80].

Overall, there is urgent need for new drugs and therapeutic targets for asthma and COPD, otherwise we will be treating only disease symptoms, with very limited ability to change disease trajectory [11,69,81,82]. Further, more research is needed on how we can decrease the risk of lung cancer and small airway destruction in smokers and patients with COPD [83]. Better management of COVID-19 in these patient populations is crucial [75,84]. Great caution is required with electronic cigarettes and other heat-not-burn tobacco products, as they are detrimental to lung health [85]. As Pulmonology Section Editor-in-Chief, I am very thankful to the authors, our reviewers and the *JCM* team for their support. I hope you will continue to consider *JCM* as the preferred home for your manuscripts.

## References

[1] Jung Y., Wee J.H., Kim J.-H., Choi H.G. (2021). The Effects of Previous Asthma and COPD on the Susceptibility to and Severity of COVID-19: A Nationwide Cohort Study in South Korea. J. Clin. Med..

[2] Haug G., Eapen M.S., Sohal S.S. (2020). Renin-Angiotensin-Aldosterone System Inhibitors in Covid-19. N. Engl. J. Med..

[3] van Zyl-Smit R.N., Richards G., Leone F.T. (2020). Tobacco smoking and COVID-19 infection. Lancet Respir. Med..

[4] Clift A.K., von Ende A., Tan P.S., Sallis H.M., Lindson N., Coupland C.A.C., Munafò M.R., Aveyard P., Hippisley-Cox J., Hopewell J.C. (2021). Smoking and COVID-19 outcomes: An observational and Mendelian randomisation study using the UK Biobank cohort. Thorax.

[5] Asrani P., Eapen M.S., Hassan M.I., Sohal S.S. (2021). Implications of the second wave of COVID-19 in India. Lancet Respir. Med..

[6] Asrani P., Tiwari K., Eapen M.S., Hassan M.D.I., Sohal S.S. (2022). Containment strategies for COVID-19 in India: Lessons from the second wave. Expert Rev. Anti-Infect. Ther..

[7] Brake S.J., Barnsley K., Lu W., McAlinden K.D., Eapen M.S., Sohal S.S. (2020). Smoking Upregulates Angiotensin-Converting Enzyme-2 Receptor: A Potential Adhesion Site for Novel Coronavirus SARS-CoV-2 (Covid-19). J. Clin. Med..

[8] Radzikowska U., Ding M., Tan G., Zhakparov D., Peng Y., Wawrzyniak P., Wang M., Li S., Morita H., Altunbulakli C. (2020). Distribution of ACE2, CD147, CD26, and other SARS-CoV-2 associated molecules in tissues and immune cells in health and in asthma, COPD, obesity, hypertension, and COVID-19 risk factors. Allergy.

[9] Finney L.J., Glanville N., Farne H., Aniscenko J., Fenwick P., Kemp S.V., Trujillo-Torralbo M.-B., Loo S.L., Calderazzo M.A., Wedzicha J.A. (2021). Inhaled corticosteroids downregulate the SARS-CoV-2 receptor ACE2 in COPD through suppression of type I interferon. J. Allergy Clin. Immunol..

[10] Higham A., Singh D. (2020). Increased ACE2 Expression in Bronchial Epithelium of COPD Patients who are Overweight. Obesity.

[11] Brake S.J., Eapen M.S., McAlinden K.D., Markos J., Haug G., Larby J., Chia C., Hardikar A., Singhera G.K., Hackett T.L. (2022). SARS-CoV-2 (COVID-19) Adhesion Site Protein Upregulation in Small Airways, Type 2 Pneumocytes, and Alveolar Macrophages of Smokers and COPD—Possible Implications for Interstitial Fibrosis. Int. J. Chron. Obstruct. Pulmon. Dis..

[12] Lu W., Eapen M.S., Singhera G.K., Markos J., Haug G., Chia C., Larby J., Brake S.J., Westall G.P., Jaffar J. (2022). Angiotensin-Converting Enzyme 2 (ACE2), Transmembrane Peptidase Serine 2 (TMPRSS2), and Furin Expression Increases in the Lungs of Patients with Idiopathic Pulmonary Fibrosis (IPF) and Lymphangioleiomyomatosis (LAM): Implications for SARS-CoV-2 (COVID-19) Infections. J. Clin. Med..

[13] Eapen M.S., Lu W., Gaikwad A.V., Bhattarai P., Chia C., Hardikar A., Haug G., Sohal S.S. (2020). Endothelial to mesenchymal transition: A precursor to post-COVID-19 interstitial pulmonary fibrosis and vascular obliteration?. Eur. Respir. J..

[14] Eapen M.S., Lu W., Hackett T.L., Singhera G.K., Thompson I.E., McAlinden K.D., Hardikar A., Weber H.C., Haug G., Wark P.A.B. (2021). Dysregulation of endocytic machinery and ACE2 in small airways of smokers and COPD patients can augment their susceptibility to SARS-CoV-2 (COVID-19) infections. Am. J. Physiol. Lung Cell. Mol. Physiol..

[15] Eapen M.S., Hansbro P.M., McAlinden K., Kim R.Y., Ward C., Hackett T.L., Walters E.H., Sohal S.S. (2017). Abnormal M1/M2 macrophage phenotype profiles in the small airway wall and lumen in smokers and chronic obstructive pulmonary disease (COPD). Sci. Rep..

[16] Eapen M.S., Sohal S.S. (2018). Understanding novel mechanisms of microbial pathogenesis in chronic lung disease: Implications for new therapeutic targets. Clin. Sci..

[17] Hönzke K., Obermayer B., Mache C., Fathykova D., Kessler M., Dökel S., Wyler E., Baumgardt M., Löwa A., Hoffmann K. (2022). Human lungs show limited permissiveness for SARS-CoV-2 due to scarce ACE2 levels but virus-induced expansion of inflammatory macrophages. Eur. Respir. J..

[18] Wark P.A.B., Pathinayake P.S., Kaiko G., Nichol K., Ali A., Chen L., Sutanto E.N., Garratt L.W., Sohal S.S., Lu W. (2021). ACE2 expression is elevated in airway epithelial cells from older and male healthy individuals but reduced in asthma. Respirology.

[19] Wark P.A.B., Pathinayake P.S., Eapen M.S., Sohal S.S. (2021). Asthma, COPD and SARS-CoV-2 infection (COVID-19): Potential mechanistic insights. Eur. Respir. J..

[20] McAlinden K.D., Eapen M.S., Lu W., Chia C., Haug G., Sohal S.S. (2020). COVID-19 and vaping: Risk for increased susceptibility to SARS-CoV-2 infection?. Eur. Respir. J..

[21] McAlinden K.D., Eapen M.S., Lu W., Sharma P., Sohal S.S. (2020). The rise of electronic nicotine delivery systems and the emergence of electronic-cigarette-driven disease. Am. J. Physiol. Lung Cell. Mol. Physiol..

[22] McAlinden K.D., Barnsley K., Weber H.C., Haug G., Chia C., Eapen M.S., Sohal S.S. (2021). Cochrane review update leaves big questions unanswered regarding vaping: Implications for medical practitioners. Eur. Respir. J..

[23] McAlinden K.D., Lu W., Eapen M.S., Sohal S.S. (2021). Electronic cigarettes: Modern instruments for toxic lung delivery and posing risk for the development of chronic disease. Int. J. Biochem. Cell Biol..

[24] McAlinden K.D., Lu W., Ferdowsi P.V., Myers S., Markos J., Larby J., Chia C., Weber H.C., Haug G., Eapen M.S. (2021). Electronic Cigarette Aerosol Is Cytotoxic and Increases ACE2 Expression on Human Airway Epithelial Cells: Implications for SARS-CoV-2 (COVID-19). J. Clin. Med..

[25] McAlinden K.D., Eapen M.S., Lu W., Sharma P., Sohal S.S. (2020). The Ill Effects of IQOS on Airway Cells: Let’s Not Get Burned All Over Again. Am. J. Respir. Cell Mol. Biol..

[26] Sohal S.S., Eapen M.S., Naidu V.G.M., Sharma P. (2019). IQOS exposure impairs human airway cell homeostasis: Direct comparison with traditional cigarette and e-cigarette. ERJ Open Res..

[27] Serpa G.L., Renton N.D., Lee N., Crane M.J., Jamieson A.M. (2020). Electronic Nicotine Delivery System Aerosol-induced Cell Death and Dysfunction in Macrophages and Lung Epithelial Cells. Am. J. Respir. Cell Mol. Biol..

[28] Eapen M.S., Sharma P., Sohal S.S. (2019). Mitochondrial dysfunction in macrophages: A key to defective bacterial phagocytosis in COPD. Eur. Respir. J..

[29] Cerdán-de-Las-Heras J., Balbino F., Løkke A., Catalán-Matamoros D., Hilberg O., Bendstrup E. (2021). Effect of a New Tele-Rehabilitation Program versus Standard Rehabilitation in Patients with Chronic Obstructive Pulmonary Disease. J. Clin. Med..

[30] Huang W.-C., Fu P.-K., Chan M.-C., Chin C.-S., Huang W.-N., Lai K.-L., Wang J.-L., Hung W.-T., Wu Y.-D., Hsieh C.-W. (2021). The Long-Term Effectiveness of Omalizumab in Adult Patients with Severe Allergic Asthma: Continuous Treatment Versus Boosting Treatment. J. Clin. Med..

[31] Okayama Y., Matsumoto H., Odajima H., Takahagi S., Hide M., Okubo K. (2020). Roles of omalizumab in various allergic diseases. Allergol. Int. Off. J. Jpn. Soc. Allergol..

[32] Dantzer J.A., Wood R.A. (2018). The use of omalizumab in allergen immunotherapy. Clinical and experimental allergy. J. Br. Soc. Allergy Clin. Immunol..

[33] Dantzer J.A., Wood R.A. (2021). Update on omalizumab in allergen immunotherapy. Curr. Opin. Allergy Clin. Immunol..

[34] Pelaia C., Calabrese C., Terracciano R., de Blasio F., Vatrella A., Pelaia G. (2018). Omalizumab, the first available antibody for biological treatment of severe asthma: More than a decade of real-life effectiveness. Ther. Adv. Respir. Dis..

[35] Fu Z., Xu Y., Cai C. (2021). Efficacy and safety of omalizumab in children with moderate-to-severe asthma: A meta-analysis. J. Asthma Off. J. Assoc. Care Asthma.

[36] Colombo G.L., Di Matteo S., Martinotti C., Oselin M., Valentino M.C., Bruno G.M., Pitotti C., Menzella F. (2019). Omalizumab and long-term quality of life outcomes in patients with moderate-to-severe allergic asthma: A systematic review. Ther. Adv. Respir. Dis..

[37] Bhattarai P., Myers S., Chia C., Weber H.C., Young S., Williams A.D., Sohal S.S. (2020). Clinical Application of Forced Oscillation Technique (FOT) in Early Detection of Airway Changes in Smokers. J. Clin. Med..

[38] Mandru R., Zhou C.Y., Pauley R., Burkes R.M. (2021). Considerations for and Mechanisms of Adjunct Therapy in COPD. J. Clin. Med..

[39] Sohal S.S., Eapen M.S., Ward C., Walters E.H. (2017). Airway inflammation and inhaled corticosteroids in COPD. Eur. Respir. J..

[40] Eapen M.S., Myers S., Walters E.H., Sohal S.S. (2017). Airway inflammation in chronic obstructive pulmonary disease (COPD): A true paradox. Expert Rev. Respir. Med..

[41] Eapen M.S., McAlinden K., Tan D., Weston S., Ward C., Muller H.K., Walters E.H., Sohal S.S. (2017). Profiling cellular and inflammatory changes in the airway wall of mild to moderate COPD. Respirology.

[42] Milara J., Peiró T., Serrano A., Guijarro R., Zaragozá C., Tenor H., Cortijo J. (2014). Roflumilast N-oxide inhibits bronchial epithelial to mesenchymal transition induced by cigarette smoke in smokers with COPD. Pulm. Pharmacol. Ther..

[43] Milara J., Peiró T., Serrano A., Artigues E., Aparicio J., Tenor H., Sanz C., Cortijo J. (2015). Simvastatin Increases the Ability of Roflumilast N-oxide to Inhibit Cigarette Smoke-Induced Epithelial to Mesenchymal Transition in Well-differentiated Human Bronchial Epithelial Cells in vitro. COPD J. Chronic Obstr. Pulm. Dis..

[44] Sohal S.S. (2015). Chronic Obstructive Pulmonary Disease (COPD) and Lung Cancer: Epithelial Mesenchymal Transition (EMT), the Missing Link?. EBioMedicine.

[45] Eapen M.S., Lu W., Hackett T.L., Singhera G.K., Mahmood M.Q., Hardikar A., Ward C., Walters E.H., Sohal S.S. (2021). Increased myofibroblasts in the small airways, and relationship to remodelling and functional changes in smokers and COPD patients: Potential role of epithelial-mesenchymal transition. ERJ Open Res..

[46] Lu W., Sharma P., Eapen M.S., Sohal S.S. (2019). Inhaled corticosteroids attenuate epithelial mesenchymal transition: Implications for COPD and lung cancer prophylaxis. Eur. Respir. J..

[47] Sohal S., Reid D., Soltani A., Ward C., Weston S., Muller H., Wood-Baker R., Walters E.H. (2010). Reticular basement membrane fragmentation and potential epithelial mesenchymal transition is exaggerated in the airways of smokers with chronic obstructive pulmonary disease. Respirology.

[48] Sohal S.S., Reid D., Soltani A., Ward C., Weston S., Muller H.K., Wood-Baker R., Walters E.H. (2011). Evaluation of epithelial mesenchymal transition in patients with chronic obstructive pulmonary disease. Respir. Res..

[49] Sohal S.S., Soltani A., Reid D., Ward C., Wills K.E., Muller H.K., Walters E.H. (2014). A randomized controlled trial of inhaled corticosteroids (ICS) on markers of epithelial-mesenchymal transition (EMT) in large airway samples in COPD: An exploratory proof of concept study. Int. J. Chron. Obstruct. Pulmon. Dis..

[50] Soltani A., Walters E.H., Reid D.W., Shukla S.D., Nowrin K., Ward C., Muller H.K., Sohal S.S. (2016). Inhaled corticosteroid normalizes some but not all airway vascular remodeling in COPD. Int. J. Chron. Obstruct. Pulmon. Dis..

[51] Ojo O., Lagan A.L., Rajendran V., Spanjer A., Chen L., Sohal S.S., Heijink I., Jones R., Maarsingh H., Hackett T.L. (2014). Pathological changes in the COPD lung mesenchyme--novel lessons learned from in vitro and in vivo studies. Pulm. Pharmacol. Ther..

[52] Mandal S., Tejaswi T., Janivara R., Srikrishnan S., Thakur P., Sahoo S., Chakraborty P., Sohal S.S., Levine H., George J.T. (2021). Transcriptomic-Based Quantification of the Epithelial-Hybrid-Mesenchymal Spectrum across Biological Contexts. Biomolecules.

[53] Eapen M.S., Myers S., Lu W., Tanghe C., Sharma P., Sohal S.S. (2018). sE-cadherin and sVE-cadherin indicate active epithelial/endothelial to mesenchymal transition (EMT and EndoMT) in smokers and COPD: Implications for new biomarkers and therapeutics. Biomark. Biochem. Indic. Expo. Response Susceptibility Chem..

[54] Mahmood M.Q., Walters E.H., Shukla S.D., Weston S., Muller H.K., Ward C., Sohal S.S. (2017). β-catenin, Twist and Snail: Transcriptional regulation of EMT in smokers and COPD, and relation to airflow obstruction. Sci. Rep..

[55] Gaikwad A.V., Lu W., Dey S., Bhattarai P., Chia C., Larby J., Haug G., Myers S., Jaffar J., Westall G. (2022). Vascular remodelling in idiopathic pulmonary fibrosis patients and its detrimental effect on lung physiology: Potential role of endothelial-to-mesenchymal transition. ERJ Open Res..

[56] Sohal S.S. (2016). Endothelial to mesenchymal transition (EndMT): An active process in Chronic Obstructive Pulmonary Disease (COPD)?. Respir. Res..

[57] Eapen M.S., Sharma P., Gaikwad A.V., Lu W., Myers S., Hansbro P.M., Sohal S.S. (2019). Epithelial-mesenchymal transition is driven by transcriptional and post transcriptional modulations in COPD: Implications for disease progression and new therapeutics. Int. J. Chron. Obstruct. Pulmon. Dis..

[58] Eapen M.S., Sharma P., Thompson I.E., Lu W., Myers S., Hansbro P.M., Sohal S.S. (2019). Heparin-binding epidermal growth factor (HB-EGF) drives EMT in patients with COPD: Implications for disease pathogenesis and novel therapies. Lab. Investig. A J. Tech. Methods Pathol..

[59] Eapen M.S., Sohal S.S. (2020). WNT/β-catenin pathway: A novel therapeutic target for attenuating airway remodelling and EMT in COPD. EBioMedicine.

[60] Jolly M.K., Ward C., Eapen M.S., Myers S., Hallgren O., Levine H., Sohal S.S. (2018). Epithelial-mesenchymal transition, a spectrum of states: Role in lung development, homeostasis, and disease. Dev. Dyn. Off. Publ. Am. Assoc. Anat..

[61] Albert R.K., Connett J., Bailey W.C., Casaburi R., Cooper J.A.D., Criner G.J., Curtis J.L., Dransfield M.T., Han M.K., Lazarus S.C. (2011). Azithromycin for prevention of exacerbations of COPD. N. Engl. J. Med..

[62] Banjanac M., Kos V.M., Nujić K., Vrančić M., Belamarić D., Crnković S., Hlevnjak M., Haber V.E. (2012). Anti-inflammatory mechanism of action of azithromycin in LPS-stimulated J774A.1 cells. Pharmacol. Res..

[63] Simpson J.L., Powell H., Baines K.J., Milne D., Coxson H.O., Hansbro P.M., Gibson P.G. (2014). The effect of azithromycin in adults with stable neutrophilic COPD: A double blind randomised, placebo controlled trial. PLoS ONE.

[64] Vrančić M., Banjanac M., Nujić K., Bosnar M., Murati T., Munić V., Polančec D.S., Belamarić D., Parnham M., Haber V.E. (2012). Azithromycin distinctively modulates classical activation of human monocytes in vitro. Br. J. Pharmacol..

[65] Jain S., Durugkar S., Saha P., Eapen M., Sharma P., Naidu V., Sohal S. (2020). Azithromycin Alleviates Cigarette Smoke Induced Epithelial Mesenchymal Transition (EMT), Airway Inflammation and Enhances Antioxidant Mechanisms in COPD. Am. J. Respir. Crit. Care Med..

[66] Banerjee B., Musk M., Sutanto E.N., Yerkovich S.T., Hopkins P., Knight D.A., Lindsey-Temple S., Stick S.M., Kicic A., Chambers D.C. (2012). Regional differences in susceptibiity of bronchial epithelium to mesenchymal transition and inhibition by the macrolide antibiotic azithromycin. PLoS ONE.

[67] Eapen M.S., Hansbro P.M., Larsson-Callerfelt A.-K., Jolly M.K., Myers S., Sharma P., Jones B., Rahman A., Markos J., Chia C. (2018). Chronic Obstructive Pulmonary Disease and Lung Cancer: Underlying Pathophysiology and New Therapeutic Modalities. Drugs.

[68] Gundamaraju R., Lu W., Azimi I., Eri R., Sohal S.S. (2020). Endogenous Anti-Cancer Candidates in GPCR, ER Stress, and EMT. Biomedicines.

[69] Liu G., Philp A.M., Corte T., Travis M.A., Schilter H., Hansbro N.G., Burns C.J., Eapen M.S., Sohal S.S., Burgess J.K. (2021). Therapeutic targets in lung tissue remodelling and fibrosis. Pharmacol. Ther..

[70] Atto B., Eapen M.S., Sharma P., Frey U., Ammit A.J., Markos J., Chia C., Larby J., Haug G., Weber H.C. (2019). New therapeutic targets for the prevention of infectious acute exacerbations of COPD: Role of epithelial adhesion molecules and inflammatory pathways. Clin. Sci..

[71] Sohal S.S. (2018). Inhaled corticosteroids and increased microbial load in COPD: Potential role of epithelial adhesion molecules. Eur. Respir. J..

[72] Asrani P., Eapen M.S., Chia C., Haug G., Weber H.C., Hassan I., Sohal S.S. (2020). Diagnostic approaches in COVID-19: Clinical updates. Expert Rev. Respir. Med..

[73] Asrani P., Hasan G.M., Sohal S.S., Hassan M.I. (2020). Molecular Basis of Pathogenesis of Coronaviruses: A Comparative Genomics Approach to Planetary Health to Prevent Zoonotic Outbreaks in the 21st Century. Omics A J. Integr. Biol..

[74] Asrani P., Hussain A., Nasreen K., AlAjmi M.F., Amir S., Sohal S.S., Hassan M.I. (2021). Guidelines and Safety Considerations in the Laboratory Diagnosis of SARS-CoV-2 Infection: A Prerequisite Study for Health Professionals. Risk Manag. Healthc. Policy.

[75] Asrani P., Tiwari K., Eapen M.S., McAlinden K.D., Haug G., Johansen M.D., Hansbro P.M., Flanagan K.L., Hassan I., Sohal S.S. (2022). Clinical features and mechanistic insights into drug repurposing for combating COVID-19. Int. J. Biochem. Cell Biol..

[76] Kumari P., Singh A., Ngasainao M.R., Shakeel I., Kumar S., Lal S., Singhal A., Sohal S., Singh I.K., Hassan M. (2020). Potential diagnostics and therapeutic approaches in COVID-19. Clin. Chim. Acta.

[77] Grigg J., Walters H., Sohal S.S., Wood-Baker R., Reid D.W., Xu C.B., Edvinsson L., Morissette M.C., Stämpfli M.R., Kirwan M. (2012). Cigarette smoke and platelet-activating factor receptor dependent adhesion of Streptococcus pneumoniae to lower airway cells. Thorax.

[78] Miyashita L., Suri R., Dearing E., Mudway I., E Dove R., Neill D., Van Zyl-Smit R., Kadioglu A., Grigg J. (2018). E-cigarette vapour enhances pneumococcal adherence to airway epithelial cells. Eur. Respir. J..

[79] Eapen M.S., Sharma P., Moodley Y.P., Hansbro P.M., Sohal S.S. (2019). Dysfunctional Immunity and Microbial Adhesion Molecules in Smoking-induced Pneumonia. Am. J. Respir. Crit. Care Med..

[80] Sohal S.S. (2017). Fluticasone propionate and increased risk of pneumonia in COPD: Is it PAFR-dependent?. Int. J. Chron. Obstruct. Pulmon. Dis..

[81] Eapen M.S., Sohal S.S. (2019). Update on the Pathogenesis of COPD. N. Engl. J. Med..

[82] Sohal S.S., Hansbro P.M., Walters E.H. (2017). Epithelial Mesenchymal Transition in Chronic Obstructive Pulmonary Disease, a Precursor for Epithelial Cancers: Understanding and Translation to Early Therapy. Ann. Am. Thorac. Soc..

[83] Sohal S.S., Walters E.H. (2017). Advanced Non–Small-Cell Lung Cancer. N. Engl. J. Med..

[84] Shinde T., Hansbro P.M., Sohal S.S., Dingle P., Eri R., Stanley R. (2020). Microbiota Modulating Nutritional Approaches to Countering the Effects of Viral Respiratory Infections Including SARS-CoV-2 through Promoting Metabolic and Immune Fitness with Probiotics and Plant Bioactives. Microorganisms.

[85] McAlinden K.D., Sohal S.S., Sharma P. (2019). There can be smoke without fire: Warranted caution in promoting electronic cigarettes and heat not burn devices as a safer alternative to cigarette smoking. ERJ Open Res..

